# 3D Zero Poisson’s Ratio Honeycomb Structure for Morphing Wing Applications

**DOI:** 10.3390/biomimetics7040198

**Published:** 2022-11-12

**Authors:** Xiaobo Gong, Chengwei Ren, Jian Sun, Peiru Zhang, Lei Du, Fang Xie

**Affiliations:** 1 School of Ocean Engineering, Harbin Institute of Technology, Weihai 264209, China; 2 National Key Laboratory of Science and Technology on Advanced Composites in Special Environments, Harbin Institute of Technology, Harbin 150001, China; 3 School of Materials Science and Engineering, Harbin Institute of Technology, Weihai 264209, China

**Keywords:** zero Poisson’s ratio, ZPR, morphing structure, 3D ZPR, adaptive morphing structure

## Abstract

Such as flying creatures, morphing aircraft can expand their aerodynamic flight envelopes by changing aerodynamic shapes, significantly improving the scope of application and flight efficiency. A novel 3D Zero Poisson’s Ratio (ZPR) honeycomb structure is designed to meet the flexible deformation requirements of morphing aircraft. The 3D ZPR honeycomb can deform in the three principal directions with smooth borders and isotropic. Analytical models related to the uniaxial and shear stiffnesses are derived using the Timoshenko beam model and validated using the quasi-static compression test. The Poisson’s ratio of the 3D ZPR honeycomb structure has an average value of 0.0038, proving the feasibility of the 3D ZPR concept. Some pneumatic muscle fibers are introduced into the system as flexible actuators to drive the active deformation of the 3D ZPR honeycomb. The active 3D ZPR honeycomb can contract by 14.4%, unidirectionally bend by 7.8°, and multi-directions bend under 0.4 Mpa pressure. Both ZPR properties and flexible morphing capabilities show the potential of this novel 3D ZPR configuration for morphing wings.

## 1. Introduction

Conventional fixed-wing aircraft generally have the best aerodynamic efficiency in a single flight condition and cannot have the best aerodynamic efficiency in the entire flight envelope. Such as flying creatures such as birds, bats, and insects in nature, morphing aircraft can expand their aerodynamic flight envelopes by changing aerodynamic shapes, significantly improving flight efficiency and scope, and performing multiple tasks [1]. As a cutting-edge technology in the modern aerospace field, morphing aircraft technology is a significant development direction for future aircraft [2,3]. A morphing vehicle is a complex system [4,5], which should balance the flexibility (performing wing deformation), stiffness (withstanding aerodynamic loads), and weight (minimizing airframe weight and maximizing payload) for optimal performance [6,7].

The honeycomb structure’s lightweight and high out-of-plane stiffness make it an ideal material for morphing aircraft [8]. Olympio and Gandhi [9] first adopted the honeycomb composite structure composed of deformed core material and flexible skin as a morphing structure. The hexagonal honeycomb structure originated from nature and has been widely used in engineering, and its elastic and nonlinear mechanical properties have been studied in detail through theoretical analysis, numerical analysis, and experimental verification [10,11]. Based on the traditional Positive Poisson’s ratios (PPR) hexagonal honeycomb, researchers have proposed a variety of novel honeycomb metamaterials exhibiting Negative Poisson’s ratios (NPR) and Zero Poisson’s ratios (ZPR). The topological shape of NPR honeycombs includes re-entrant hexagonal honeycombs [10,12,13], chiral honeycombs [14,15,16], star honeycombs [17,18], and double-arrow honeycombs [19]. When a PZR/NZR honeycomb is stretched in one direction, it shrinks/expands in the orthogonal directions [20]. If the deformation in the non-loading direction is constrained, the equivalent stiffness of the honeycomb structure in the loading direction increases, increasing the driving force demand. On the other hand, when a PPR/NPR honeycomb is bent out-of-plane, the honeycombs will warp, with the PPR honeycomb appearing as a saddle shape and NPR honeycombs appearing hyperbolic [11,21].

To overcome the shortcomings of the PPR/NPR honeycomb and meet the deformation requirements of the morphing structure, researchers have designed a variety of ZPR honeycombs [22,23,24,25,26], such as accordion honeycombs [27,28,29], PZP-NZP hybrid honeycomb [20], SILICOMB honeycombs [23,30], fish cell honeycomb [31], chiral cellular structure [32], four-pointed star shape honeycombs [26] and reconfigurable mechanism modules structures [33]. These ZPR honeycombs have been well studied and explored for the initial application of morphing skin [20,26,27,34,35]. Among them, the four-pointed star shape honeycomb can deform in two orthogonal directions in-plane and avoid out-of-plane warping deformation. Various optimized honeycombs are then proposed to improve further the deformation ability of the four-pointed star honeycomb structure. The honeycomb arms are upgraded from straight lines to sine and cosine curves [24,25,36]. Currently, most research on ZPR honeycombs focuses on two-dimensional topological shapes and the analysis of basic mechanical properties [37,38,39], while there are relatively few studies on three-dimensional honeycomb structures. With the rapid development of 3D printing technology, rapid additive manufacturing of complex-shaped components has become a reality. Researchers have designed and fabricated a variety of 3D NPR honeycomb superstructures [37,40,41,42] by arranging the 2D lattices appropriately, such as 3D chiral [43], 3D re-entrant [18], and 3D rotating chiral [37,38]. However, 3D ZPR honeycombs are rarely reported in the literature, and 2D ZPR honeycombs cannot meet the demands of morphing aircraft for complex adaptive morphing structures.

This paper proposes a novel 3D ZPR honeycomb based on the four-pointed star-shaped honeycomb topology. The 3D ZPR honeycomb can deform in the three principal directions, and the honeycomb boundary is smooth and continuous and has good isotropy in the whole deformation process. The elasticity of this 3D ZPR honeycomb is derived from a theoretical analysis model and validated by experiments. Furthermore, some McKibben pneumatic muscle fibers are introduced into the system as flexible actuators to drive the active deformation of the 3D ZPR honeycomb. 3D ZPR honeycombs can conduct contraction and multidirectional bending deformation activated by pneumatic muscle fiber, which provides a new choice for morphing wing structure.

**Figure 1:**
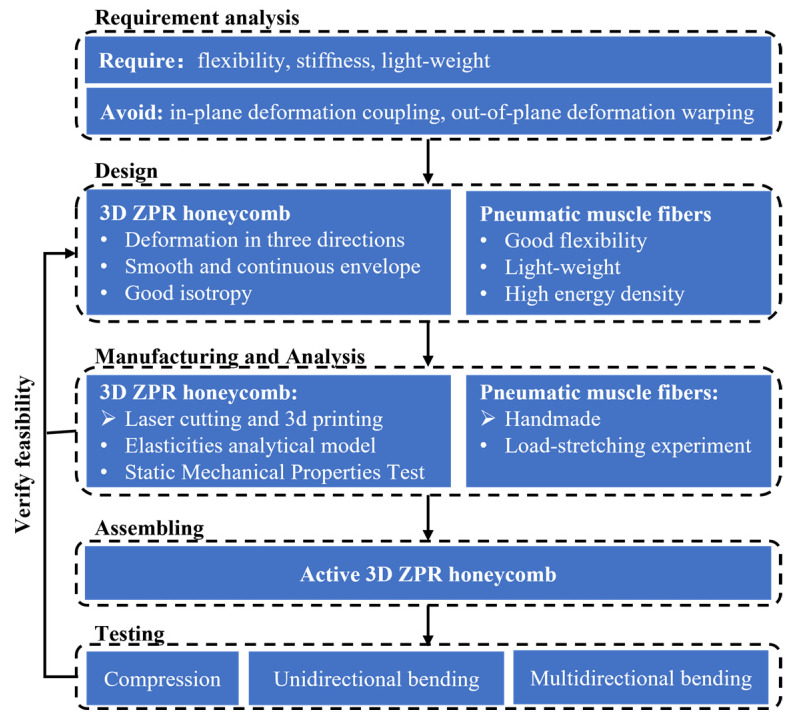
The structure and organization of the paper.

The rest of this paper is organized as follows, as shown in Figure 1. Section 1 reviews the zero Poisson’s ratio honeycomb and its application in morphing aircraft and condenses the design requirements of the morphing structure. According to the requirements, a novel 3D ZPR honeycomb structure is designed, and pneumatic muscle fibers are proposed as actuators to drive the active deformation of the 3D ZPR honeycomb in Section 2. The elasticity analytical model of this 3D ZPR honeycomb is derived in Section 3 and validated by experiments in Section 4. Finally, Section 5 integrates the honeycomb and pneumatic muscle fibers actuators into an active 3D ZPR honeycomb and conforms deformation tests to verify the feasibility of the design.

## 2. 3D ZPR Honeycomb Design

**Figure 2:**
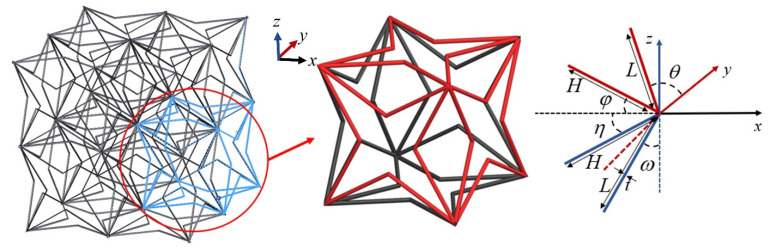
The layout of the novel zero Poisson’s ratio honeycomb and geometric parameters of a unit cell.

The layout of the novel 3D ZPR honeycomb and its unit cell are presented in Figure 2. The 3D ZPR honeycomb structure consists of hexahedral unit cells composed of six four-pointed star-shaped 2D structures. Defining two walls express the geometry of four-pointed star-shaped lattices with lengths *H* and *L*, thickness *t*, heights *b,* and two slope angles *θ* and *φ* in the *x-o-y* plane, and two slope angles *η* and *ω* in the *x-o-z* plane. The length parameters can also express dimensionless by *α, β*, and *γ*, where *α = H/L, β = t/L, and γ = b/L.*

## 3. Elasticities Analytical Model

**Figure 3:**
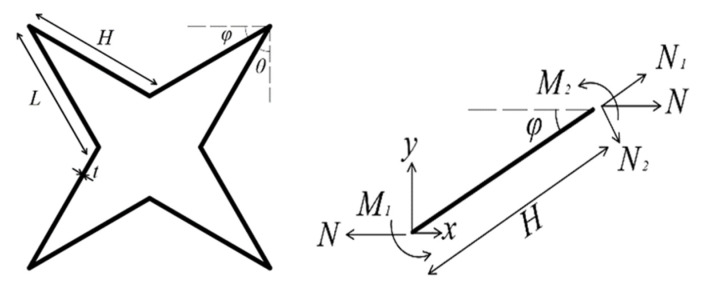
Forces and moments correspond to the homogenized *x* direction tensile properties.

The 3D ZPR honeycomb is composed of four-pointed star-shaped unit elements. The four-pointed star-shaped structure’s elasticities are theoretically analyzed using the Timoshenko beam model, and the calculation diagram is shown in Figure 3. When a stress *σ_x_* is applied in the *x* direction, one set of cell walls along the *x*-direction carries the load. By symmetry, the quarter unit cell is considered with two fixed ends and loaded along the *x*-direction.

By the moment balance, we can obtain (1)~(6):

(1) $$N=bl\cos\theta \sigma_{x}$$

(2) $$N_{1}=N\cos\varphi$$

(3) $$N_{2}=N\sin\varphi$$

(4) $$M(0)=M_{1}=\frac{NH\sin\varphi }{2}$$

(5) $$M(H\cos\varphi )=M_{2}=-\frac{NH\sin\varphi }{2}$$

(6) $$M(x)=NH\sin\varphi (\frac{1}{2}-\frac{x}{H\cos\varphi })$$

Using Castiglino’s Theorem, the deflection is equal to the partial derivative of strain energy, and we can obtain the deflection *δ_x_*.

(7) $$\delta_{x}=\int \frac{M}{E_{s}I}\frac{\partial M}{\partial N}ds+\int \frac{N_{1}}{E_{s}A}\frac{\partial N_{1}}{\partial N}ds+\int \kappa \frac{N_{2}}{G_{s}A}\frac{\partial N_{2}}{\partial N}ds$$

(8) $$G_{s}=\frac{E_{s}}{2(\nu +1)}$$

where the section moment of inertia of the honeycomb wall is expressed by *I* = *bt*^3^/12, the shear stress shape coefficient of the rectangular section *κ* is 1.2, d*s* = d*x*/cos*φ*.

The average strain in the *x*-direction can be expressed as:

(9) $$\varepsilon_{x}=\frac{\delta_{x}}{H\cos\varphi }$$

The equivalent elastic modulus in the *x*-direction is:

(10) $$E_{x}=\frac{\sigma_{x}}{\varepsilon_{x}}$$

The nondimensional elastic modulus *E_x_/E_s_* in the *x*-direction is dimensionless, and the final result can be obtained as shown in the formula (11).

(11) $$\frac{E_{x}}{E_{s}}=\frac{\beta^{3}}{\cos\theta [(\alpha^{2}+3.12\beta^{2})\sin\varphi \tan\varphi +\beta^{2}\cos\varphi ]}$$

A similar methodology can obtain nondimensional elastic modulus *E_y_/E_s_* and *E_z_/E_s_* along the *y*-direction and *z*-direction, respectively.

(12) $$\frac{E_{y}}{E_{s}}=\frac{\beta^{3}\cos\theta }{\alpha \cos\varphi [(1+3.12\beta^{2})\sin^{3}\theta +\beta^{2}\sin\theta \cos^{2}\theta ]}$$

(13) $$\frac{E_{z}}{E_{s}}=\frac{\beta^{3}\cos\omega }{\alpha \cos\eta [(1+3.12\beta^{2})\sin^{3}\omega +\beta^{2}\sin\omega \cos^{2}\omega ]}$$

The 3D ZPR honeycomb structure comprises an orthogonal arrangement of four-pointed star structure lattices. Due to symmetry, the rotation at the intersection of the cell arms is zero. By ignoring the rotation deformation at the intersection, the 3D ZPR honeycomb has independent deformations along the *x*, *y*, and *z* directions. So the Poisson’s ratio *υ_xy_*, *υ_yz,_* and *υ_zx_* are zero.

**Figure 4:**
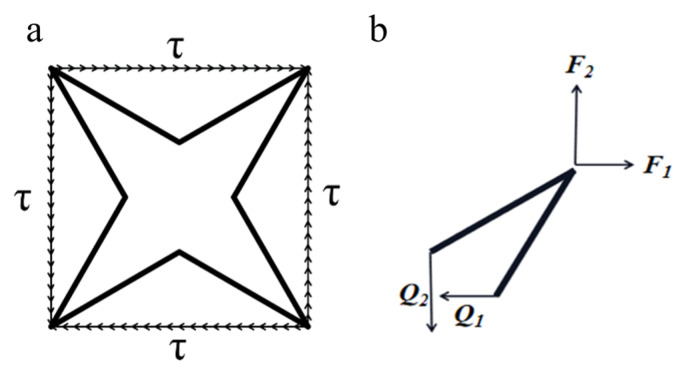
The four-pointed star honeycomb in pure shear: (**a**) unit cell subjected to pure shear stress, (**b**) the loads and displacements of the quarter unit cell caused by shear stress.

When the four-pointed star structure is subjected to uniform, pure shear stress, as shown in Figure 4, the anti-symmetry of the system is used to select 1/4 of the cells for analysis. There are only anti-symmetric internal forces on the symmetrical plane, and the symmetrical internal forces are zero, so it can be concluded that only anti-symmetric shear forces (*Q_1_* and *Q_2_*) exist on the symmetrical plane.

The loads can be determined from the equilibrium of the quarter unit cell, for which:

(14) $$F_{1}=\tau H\cos\varphi$$

(15) $$F_{2}=\tau L\cos\theta$$

(16) $$Q_{1}=F_{1}$$

(17) $$Q_{2}=F_{2}$$

The displacements Δ*_x_* and Δ*_y_* at the cell arms endpoints are derived as:

(18) $$\Delta_{x}=\frac{Q_{1}L^{3}\cos^{2}\theta }{3E_{s}I}+\frac{Q_{1}L\sin^{2}\theta }{E_{s}A}$$

(19) $$\Delta_{y}=\frac{Q_{2}H^{3}\cos^{2}\varphi }{3E_{s}I}+\frac{Q_{2}H\sin^{2}\varphi }{E_{s}A}$$

The shear strain *γ_xy_* and shear modulus *G_xy_* is therefore obtained as:

(20) $$\gamma_{xy}=\frac{\Delta_{x}}{L\cos\theta }+\frac{\Delta_{y}}{H\cos\varphi }$$

(21) $$G_{xy}=\frac{\tau }{\gamma_{xy}}$$

The non-dimensional shear modulus is finally expressed as:

(22) $$\frac{G_{xy}}{E_{s}}=\frac{\beta^{3}}{4\alpha (\alpha +1)\cos\theta \cos\varphi +\beta^{2}(\alpha \cos\varphi \sin\theta \tan\theta +\cos\theta \sin\varphi \tan\varphi )}$$

A similar methodology can obtain equivalent shear modulus *G_xz_*:

(23) $$\frac{G_{xz}}{E_{s}}=\frac{\beta^{3}}{4\alpha (\alpha +1)\cos\omega \cos\eta +\beta^{2}(\alpha \cos\eta \sin\omega \tan\omega +\cos\omega \sin\eta \tan\eta )}$$

## 4. Static Mechanical Properties Test

### 4.1. Young’s Modulus Test

**Figure 5:**
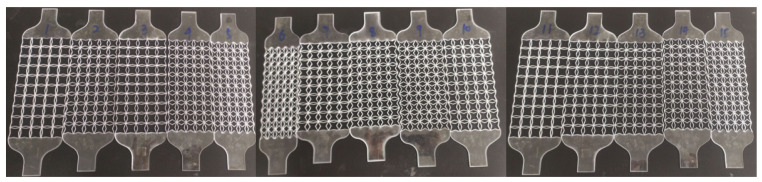
Four-pointed star honeycomb experimental specimen.

A laser cutting machine was used to cut the polymethyl methacrylate (PMMA) sheet to obtain the test specimen, as shown in Figure 5. The equipment used in this paper’s tension and compression experiments is a WDW-50 universal testing machine (Jinan east testing machine Co. Ltd., Jinan, China). First, the elastic modulus of PMMA was obtained by a quasi-static tensile experiment according to the standard GB/T1447-2005. Young’s modulus of the PMMA is 2685 MPa. Fifteen groups of typical specimens were selected for testing. The dimensions of each specimen are shown in Table 1, where *H* = *L* = 10 mm, *α* = 1, *β* = 0.2, and the value ranges of *θ* and *φ* are 10–50°. Three specimens were selected for each geometric configuration, and the final result was taken as the average of the three.

**Figure 6:**
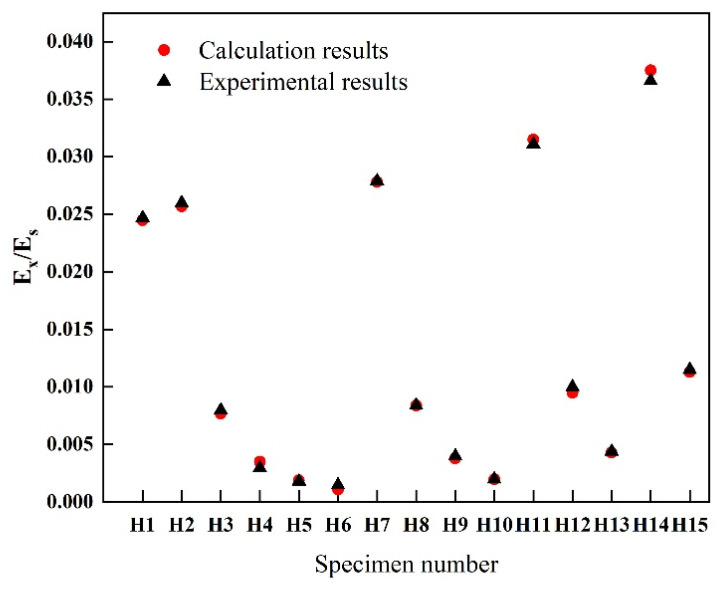
Experimental and theoretical predictions for the nondimensional Young’s modulus.

The experimental and theoretical values of the uniaxial tensile Young’s modulus of the four-pointed star structure are shown in Figure 6. As shown, the experimental results agree with the theoretical solutions. The relative error between the theoretical and experimental values is between 0.8% and 10%, with a mean of 5.3%. The most significant relative error occurred in the H6 sample, and the error of H14 was also relatively large. The reason is that the honeycomb arms of the experimental samples have a certain thickness. When the angle between the two arms is slight, the boundaries of the two arms will merge, resulting in errors in the length and width of the arms. The theoretical analysis adopts the beam model, and there is no cross-merge of arms. In addition, the machining accuracy of the laser cutting machine also affects the experimental results. The laser cutting path has a certain width and taper, which affects the geometry of the honeycomb arm and cannot guarantee a perfect rectangle.

### 4.2. Poisson’s Ratio Test

**Figure 7:**
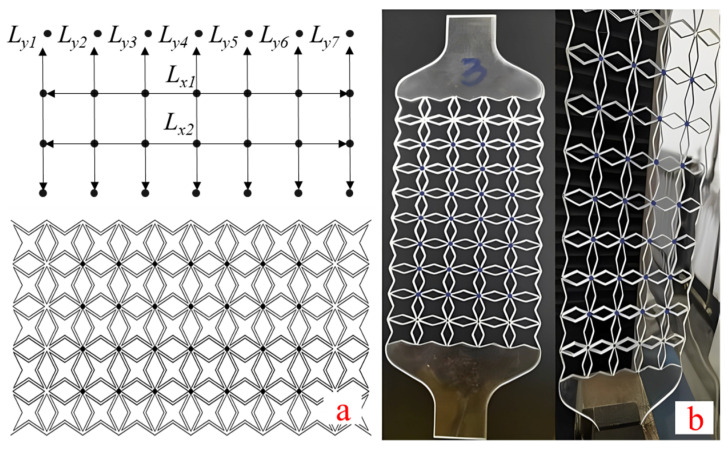
Poisson’s ratio measurement. (**a**) schematic diagram of marked points, (**b**) test sample.

The strain and Poisson’s ratio of the four-pointed star structure were measured by image measurement technology [18,43]. The number of pixels is proportional to the length, i.e., *L_A_*_−*B*_ ∝ *N_A_*_−*B*_, where *L_A_*_−*B*_ is the length between points A and B, and *N_A_*_−*B*_ is the number of pixels between points A and B. The strain can be measured by the variation in the number of pixels. Herein, 28 points were marked on the honeycomb structure with a marker pen, as shown in Figure 7.

The following formulas calculated the strain and Poisson’s ratio:

(24) $$\varepsilon_{y}=\frac{\sum_{i=1}^{7}L_{yi}^{\prime }/7-\sum_{i=1}^{7}L_{yi}/7}{\sum_{i=1}^{7}L_{yi}/7}$$

(25) $$\varepsilon_{x}=\frac{\sum_{i=1}^{2}L_{xi}^{\prime }/2-\sum_{i=1}^{2}L_{xi}/2}{\sum_{i=1}^{2}L_{xi}/2}$$

*L’_xi_* and *L’_yi_* are the lengths between the marker points in the *x*-direction and *y*-direction after deformation, respectively; *L_xi_* and *L_yi_* are the original lengths between the marker points in the x-direction and *y*-direction, respectively.

(26) $$\upsilon_{xy}=-\frac{\varepsilon_{y}}{\varepsilon_{x}}$$

**Figure 8:**
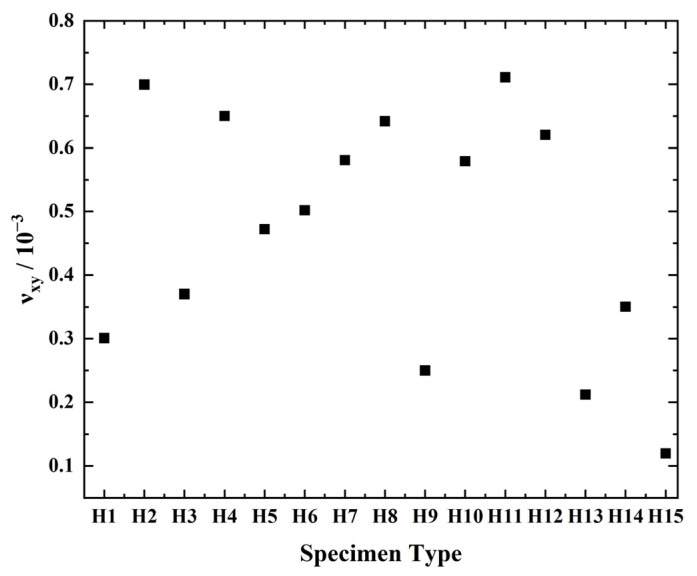
Experimental test results of Poisson’s ratio of four-pointed star structure.

During the tensile process of the four-pointed star structure, photos were taken to record the deformation. The distance between two points in the image is calculated by the Euclidean Distance method in the pixel coordinate system. The test results of Poisson’s ratio are shown in Figure 8. It can be seen that the maximum Poisson ratio of the four-pointed star honeycomb structure is 0.7 × 10^−3^, which is almost 0, which can confirm the ZPR character.

### 4.3. 3D ZPR Honeycomb Compression Test

**Figure 9:**
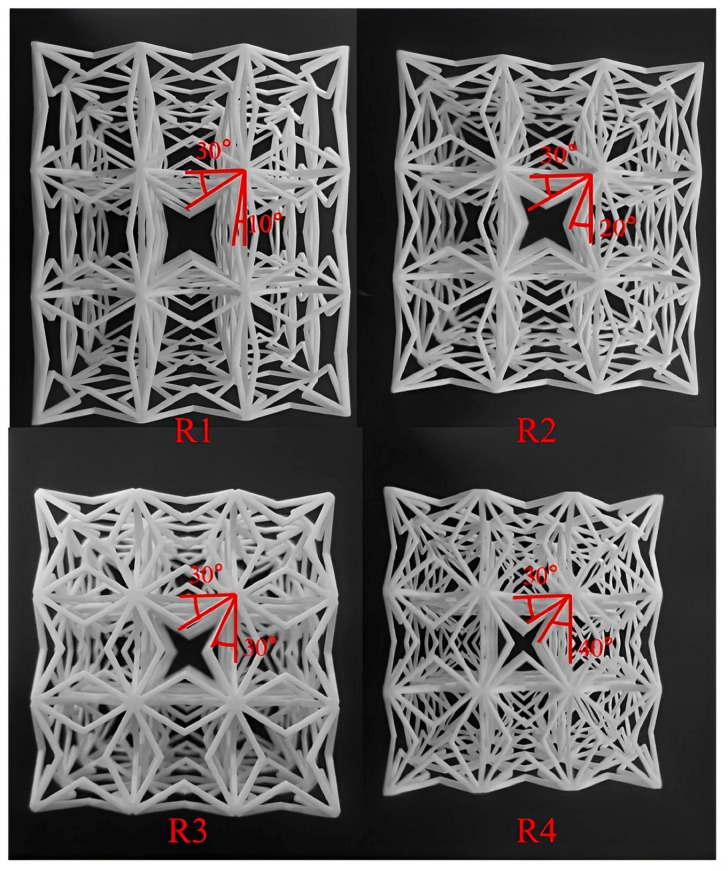
3D ZPR honeycomb specimen for compression test.

The compression test samples containing 3 × 3 × 3 hexahedral unit cells were prepared using Selective Laser Sintering technology (SLS) with white nylon material (*Es* = 1150 MPa), which is shown in Figure 9. The geometric parameters of the 3D ZPR honeycomb are *H* = *L* = 17 mm, *t* = *b* = 1.5 mm, *ω* = 30°, and *η =* 10°, 20°, 30°, 40°, respectively. The compression speed was 1 mm/min.

The theoretical results for Young’s modulus can be obtained from the theoretical model in Section 2. The homogenized stress *σ_z_* in the *z* direction can be calculated using *σ_z_ = N_z_/A_z_*. Here *N_z_* is the resultant force of all honeycomb walls in the z square, represented by 48*N* (3D ZPR honeycomb samples containing 3 × 3 × 3 hexahedral unit cells have 48 cell walls in the *z*-direction); *A_z_* is the projected area of the 3D ZPR honeycomb volume envelope in the *z*-direction, *A_z_* = (6*H*cos^2^*η*)^2^. Then we obtain the expression for the homogenized stress:

(27) $$\sigma_{z}=\frac{48N}{{\left(6H\cos^{2}\eta \right)}^{2}}$$

The homogenized strain *ε_z_* in the *z* direction can be obtained using Equations (7) and (9). Substituting Equations (2)–(6) into Equation (7) yields the displacement of the monolayer honeycomb arm in the *z*-direction:

(28) $$\delta_{z}=\frac{NH\left(H^{2}\sin^{2}\omega +t^{2}\cos^{2}\omega +3.12t^{2}\sin^{2}\omega \right)}{E_{s}bt^{3}}$$

The homogenized strain *ε_z_* is finally obtained by substituting Equation (28) into Equation (9).

(29) $$\varepsilon_{z}=\frac{N\left(H^{2}\sin^{2}\omega +t^{2}\cos^{2}\omega +3.12t^{2}\sin^{2}\omega \right)}{E_{s}bt^{3}\cos\omega }$$

Then the equivalent Young’s modulus of the 3D ZPR honeycomb in the *z* direction is obtained.

(30) $$E_{z}=\frac{\sigma_{z}}{\varepsilon_{z}}=\frac{4E_{s}bt^{3}\cos\omega }{3H^{2}\cos^{2}\eta \left(H^{2}\sin^{2}\omega +t^{2}\cos^{2}\omega +3.12t^{2}\sin^{2}\omega \right)}$$

**Figure 10:**
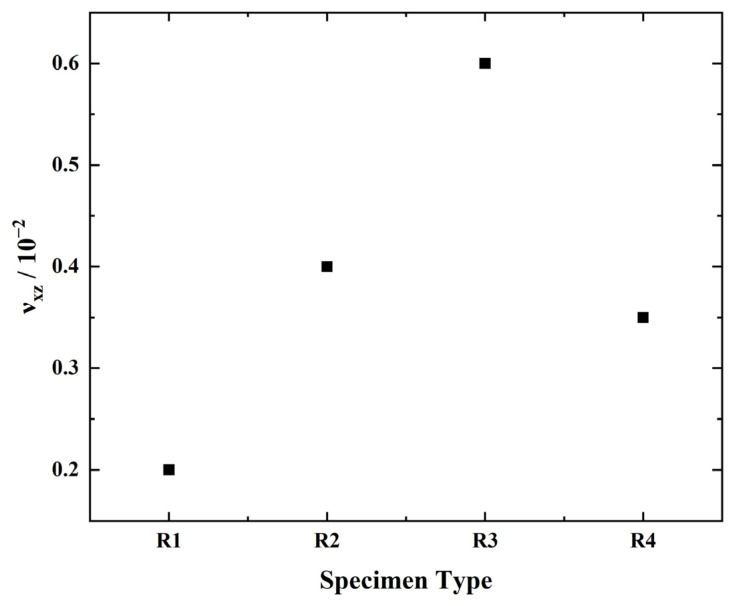
Experimental test results of Poisson’s ratio of 3D ZPR honeycomb.

The theoretical and experimental test results of Young’s modulus are listed in Table 2. The two results are in good agreement. When the angle *ω* is less than 30°, the error is less than 6%. When the angle *ω* is 40°, the error is 20.4%. The reason for the maximum error is the same as the analysis in Section 4. When *ω* is large, the two honeycomb arms are close together. At this time, the honeycomb arms with solid thickness will cross-fuse, resulting in a change in the length of the honeycomb arms. In addition, 3D printing accuracy is also an error cause. There may be slight bubble defects inside the sample, which reduces the material’s mechanical properties, which in turn causes the test results to be lower than the theoretical calculation values. The Poisson’s ratio test, as in Section 4.2, achieves the Poisson’s ratio of the 3D ZPR honeycomb, as shown in Figure 10. It can be seen that Poisson’s ratio of the 3D ZPR honeycomb is close to zero, and the average value is 3.8 × 10^−3^, which fully proves the feasibility of the ZPR design.

## 5. Design and Test of Deformation Driving Method for 3D ZPR Honeycomb

### 5.1. Active 3D ZPR Honeycomb Design

**Figure 11:**
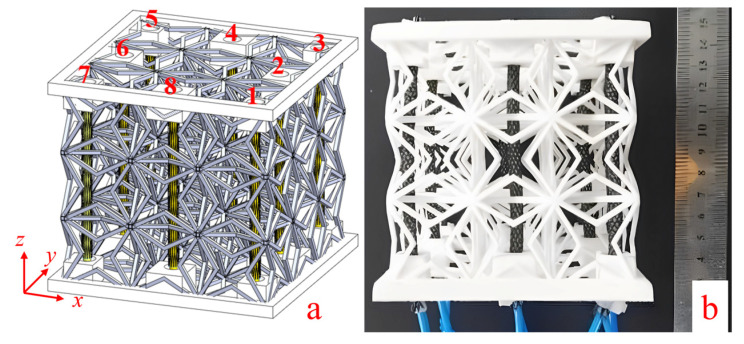
3D ZPR actuation scheme. (**a**) Concept, (**b**) Prototype.

When it is to be applied to the morphing structure, a suitable driving method is essential for the 3D ZPR honeycomb to be continuously deformed. Multiple McKibben pneumatic muscle fibers are arranged in the inner void of the 3D ZPR honeycomb structure to realize the contraction and multidirectional bending. As shown in Figure 11, eight pneumatic muscle fibers are arranged in the *z*-axis direction of the 3D ZPR honeycomb structure. The pneumatic muscle fibers undergo axial contraction under inflation, and the honeycomb structure at the installation position of the driving muscle performs compression deformation. Various forms of honeycomb deformation, such as contraction and multidirectional bending, can be achieved by programming the contraction amount of the pneumatic muscle fibers in different locations. The pneumatic muscle fiber’s inflation and deflation process control are realized by PLC (Programmable Logic Controller), controlling the opening and shutting of the pneumatic solenoid valve.

### 5.2. Pneumatic Muscle Fiber Actuation Testing

**Figure 12:**
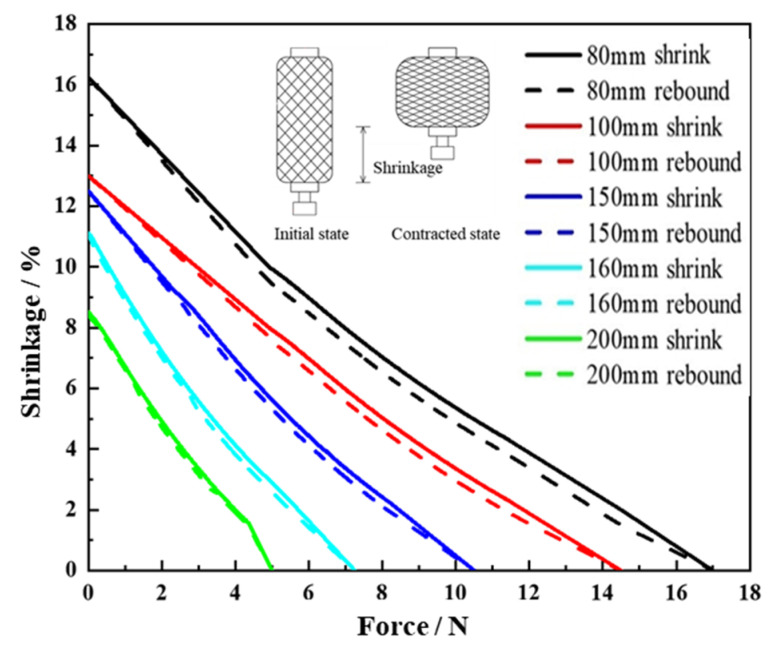
Load-stretching experiment of pneumatic muscle fibers.

The pneumatic muscle fiber is an emerging driving system that uses pneumatic energy to drive deformation, and the Mckibben type is a typical representative. The Mckibben pneumatic muscle fiber consists of a braided mesh, a rubber tube, and accessories. When the compressed air is fed into the inner rubber tube, the rubber tube begins to expand radially, increasing the braided mesh’s weaving angle and resulting in shrinkage axially. When deflated, the air pressure inside the rubber tube decreases, and the elastic force of the rubber drives the muscle to return to its original length. The deformation process is shown in Figure 12.

In this paper, the Mckibben-type pneumatic muscle fibers were fabricated by hand using Feng [44]. Pneumatic muscle fibers with lengths of 80 mm, 100 mm, 150 mm, 160 mm, and 200 mm were prepared, and their contractile properties under load were tested. The characteristic driving curve of pneumatic muscle fiber is shown in Figure 12. When the input air pressure is constant, the contraction force negatively correlates with the contraction rate. The maximum contraction rate decreases gradually with the increase in length, so the pneumatic muscle fibers with smaller lengths are selected to improve driving efficiency. To match the size of the 3D ZPR honeycomb, an 80 mm pneumatic muscle fiber is used for the deformation experiment.

### 5.3. Compression Deformation

**Figure 13:**
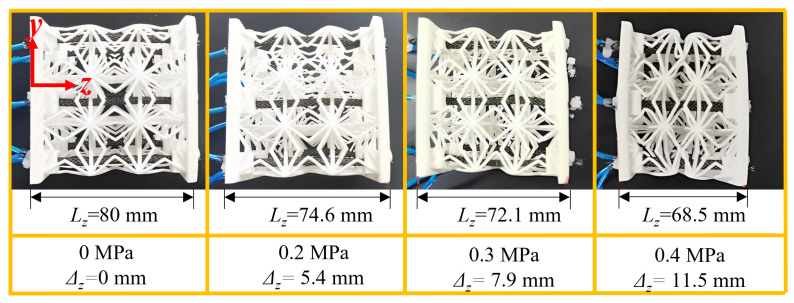
Unidirectional contraction of honeycomb under different input air pressure.

The 3D ZPR honeycomb will contract when the eight pneumatic muscle fibers are inflated simultaneously. Inflation and deflation cycles were conducted to ensure the tightness and stability of pneumatic muscle fibers. The compressed gas of 0.2 MPa, 0.3 MPa, and 0.4 MPa was simultaneously input to the pneumatic muscle, and the deformation process of the 3D ZPR honeycomb was recorded simultaneously, as shown in Figure 13. The amount of shrinkage of the 3D ZPR honeycomb increases with increasing air pressure. During the deformation process, due to the zero Poisson’s ratio characteristic of the 3D ZPR honeycomb structure, the four side borders remain flat and avoid the lateral compression/extension in response to NPR/PPR honeycomb contracting. When the pneumatic muscle fiber input pressure was 0.4 Mpa, the contraction displacement of the 3D ZPR honeycomb was 11.5 mm, 14.4% of the total length, showing a good deformation ability. The active 3D ZPR honeycomb structure has a good application prospect in the deformed airfoil with the variable span, chord, and thickness due to its good deformability and deformation decoupling in only one direction.

### 5.4. Unidirectional Bending Deformation

**Figure 14:**
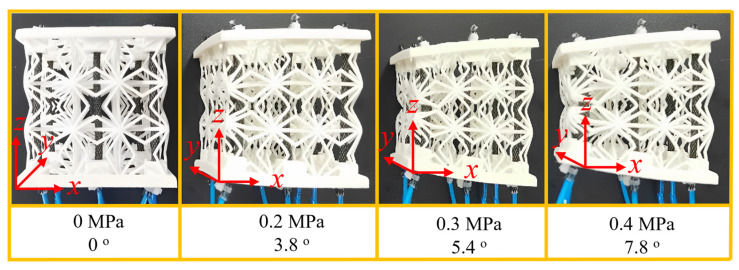
Unidirectional bending deformation of 3D ZPR honeycomb under different pressure.

When the three pneumatic muscles on the same side of the 3D ZPR honeycomb are inflated, the honeycomb structure will bend uni-direction. Similar to the deformation test process in the above section, the inflation pressure of the pneumatic muscle is 0.2 MPa, 0.3 MPa, and 0.4 Mpa, and the angles between the upper and lower faces of the honeycomb structure under different pressures are recorded, respectively. The unidirectional bending deformation of the 3D ZPR honeycomb under different air pressures is shown in Figure 14. The larger the input air pressure value, the larger the deflection angle. The deflection angles of the honeycomb structure are 3.8°, 5.4°, and 7.8° under the inflation pressure of 0.2 MPa, 0.3 MPa, and 0.4 Mpa, respectively. During the deformation process, the boundary of the honeycomb structure is smooth, and there are wrinkles, bulging, or peeling. The good unidirectional bending performance of the active 3D ZPR honeycomb structure gives it a good application prospect in the morphing wing with variable sweep angle, camber, and dihedral angle.

### 5.5. Multidirectional Bending Deformation

**Figure 15:**
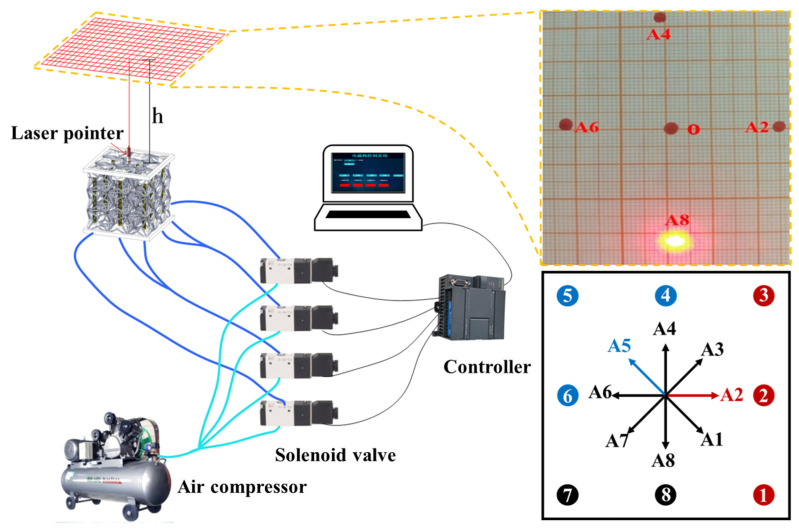
Schematic diagram of the multidirectional bending test.

Similar to unidirectional bending, more complex deformations can be achieved by adjusting the inflation position of the pneumatic muscle fibers. The inflation strategy performs various directions bending in this section. The test setup is shown in Figure 15. A laser pointer is fixed on the top face of the 3D ZPR honeycomb and deflected along with the top face to visually display the bending direction. A piece of graph paper is installed 80 cm above the laser pointer to receive the light spot, which calculates the deflection angle. Inflation strategies for pneumatic muscle fibers are listed in Table 3, where 0 means the solenoid valve is closed and the pneumatic muscle fiber is not inflated; 1 means the solenoid valve is open, the pneumatic muscle fiber is inflated, and the air pressure is 0.4 MPa. The bending direction under each inflatable deformation scheme is shown in Figure 15. As shown in Figure 15, when pneumatic muscles No. 1–3 (marked in red) are inflated simultaneously, the active 3D ZPR honeycomb structure will deflect in the direction of A2 (marked in red), and the predicted angle is 0° at this time. When pneumatic muscles No. 4–6 (marked in blue) are inflated simultaneously, the honeycomb structure will be biased towards the A5 direction (marked in blue), and the test predicted angle is 135°. The test results are consistent with the expected angle, and the error does not exceed 2.5°. This article lists eight bending directions but is not limited to only eight. More diverse bending directions and angles of the 3D ZPR honeycomb structure can be achieved by adaptively adjusting the inflation position and pressure of the pneumatic muscle fibers. Here, only the opening and shutting of the solenoid valve are used to realize the multidirectional bending of the 3D ZPR honeycomb, and the closed-loop control method can be introduced later to recognize the precise control of deformation. The flexible deformation ability of the active 3D ZPR honeycomb structure can be applied to the intelligent variable wing tip deformation wing.

## 6. Conclusions

In this work, a novel 3D ZPR honeycomb structure has been designed, modeled, and evaluated from an analytical and experimental point of view. 3D ZPR honeycomb can deform in three directions with smooth boundaries due to ZPR property, and the deformation in three movements is decoupled. The theoretical model and experiments show general agreement. The theoretical analysis model will support the practical application of 3D ZPR honeycomb and optimal design. By arranging the pneumatic muscle fibers inside the 3D ZPR honeycomb, various adaptive deformations such as unidirectional contraction, unidirectional bending, and multidirectional bending can be achieved. Various deformations of 3D ZPR honeycomb can find suitable application scenarios in morphing wings, such as contraction deformation for a variable span, chord, and thickness; unidirectional bending deformation for a variable sweep, camber, and dihedral angle; multidirectional bending deformation for variable winglet that requires more deformation freedom. The 3D ZPR honeycomb proposed in this paper is only a conceptual study, and the follow-up will carry out in-depth research on the deformation control method, the integration of honeycomb and morphing skin, and the development of morphing wing prototypes.

## Figures and Tables

**Table 1:** Specimen Geometry.

No.	H1	H2	H3	H4	H5	H6	H7	H8	H9	H10	H11	H12	H13	H14	H15
*θ*	10	20	20	20	20	20	30	30	30	30	40	40	40	50	50
*φ*	10	10	20	30	40	50	10	20	30	40	10	20	30	10	20

**Table 2:** Young’s modulus of 3D ZPR honeycomb.

Specimen No.	Specimen Size (mm)				Experiment (MPa)	Theory (MPa)	Error (%)
	*ω* (°)	*η* (°)	*H* (mm)	*L* (mm)			
R1	10	30	17	17	3.102	3.175	2.35
R2	20	30	17	17	0.891	0.919	3.14
R3	30	30	17	17	0.434	0.41	5.53
R4	40	30	17	17	0.279	0.222	20.43

**Table 3:** Pneumatic muscle fiber programming scheme for 3D ZPR multidirectional bending.

Program	Prediction (°)	1	2	3	4	5	6	7	8	Test (°)	Error (°)
A1	−45	1	1	0	0	0	0	0	1	−44	−1
A2	0	1	1	1	0	0	0	0	0	2	1.5
A3	45	0	1	1	1	0	0	0	0	47	2
A4	90	0	0	1	1	1	0	0	0	88	−2
A5	135	0	0	0	1	1	1	0	0	136	1
A6	180	0	0	0	0	1	1	1	0	178	−2
A7	225	0	0	0	0	0	1	1	1	223	−2.5
A8	270	1	0	0	0	0	0	1	1	269	−1.5

## Data Availability

Not applicable.

## Nomenclature

3D	Three-dimensional
*A*	Section area of inertia of cell wall
*b*	Wall Heights
*E_s_*	Young’s modulus of raw materials used to make honeycomb structures
*E_x_*	Equivalent elastic modulus in the *x*-direction
*E_x_/E_s_*	Nondimensional elastic modulus in the *x*-direction
*E_y_*	Equivalent elastic modulus in the *y*-direction
*E_y_/E_s_*	Nondimensional elastic modulus in the *y*-direction
*E_z_*	Equivalent elastic modulus in the *z*-direction
*E_z_/E_s_*	Nondimensional elastic modulus in the *z*-direction
*G_xy_*	Shear modulus of *x-o-y* plane
*H*	Wall length in the *x-o-y* plane
*I*	Section moment of inertia of cell wall
*L*	Wall length in the *x-o-y* plane
*L_A_*_−*B*_	Length between points A and B
*L_xi_*	Original lengths between the marker points in the *x*-direction
*L’_xi_*	Lengths between the marker points in the *x*-direction
*L_yi_*	Original lengths between the marker points in the *y*-direction
*L’_yi_*	Lengths between the marker points in the *y*-direction
*M*_1_	Bending moment at endpoint 1
*M*_2_	Bending moment at endpoint 1
*N*	Force of cell wall along the *x*-direction
*N*_1_	Force parallel to cell wall at endpoint 1
*N*_2_	Force perpendicular to cell wall at endpoint 1
*N_A_*_−*B*_	Number of pixels between points A and B
NPR	Negative Poisson’s ratios
PLC	Programmable Logic Controller
PPR	Positive Poisson’s ratios
SLS	Selective Laser Sintering technology
*t*	Wall thickness
ZPR	Zero Poisson’s Ratio
*α*	Nondimensional length, *α = H/L*
*β*	Nondimensional length, *α = t/L*
*γ*	Nondimensional length, *α = b/L*
*γ_xy_*	Shear strain in the *x-o-y* plane
*δ_x_*	Deflection under σ_x_
Δ*_x_*	Displacements in the *x*-direction at wall endpoints
Δ*_y_*	Displacements in the *y*-direction at wall endpoints
*ε_x_*	Normal strain in the *x*-direction
*ε_y_*	Normal strain in the *y*-direction
*η*	Wall slope angle in the *x-o-z* plane
*θ*	Wall slope angle in the *x-o-y* plane
*κ*	Shear stress shape coefficient of the rectangular section
*ν_xy_*	Poisson’s ratio in the *x-o-y* plane
*σ_x_*	Stress in the *x* direction
*τ*	Pure shear stress
*φ*	Wall slope angle in the *x-o-y* plane
*ω*	Wall slope angle in the *x-o-z* plane
